# Role and Posttranslational Regulation of Cx46 Hemichannels and Gap Junction Channels in the Eye Lens

**DOI:** 10.3389/fphys.2022.864948

**Published:** 2022-03-30

**Authors:** Mauricio A. Retamal, Guillermo A. Altenberg

**Affiliations:** ^1^ Universidad del Desarrollo, Centro de Fisiología Celular e Integrativa, Clínica Alemana Facultad de Medicina, Santiago, Chile; ^2^ Universidad del Desarrollo, Programa de Comunicación Celular en Cáncer, Clínica Alemana Facultad de Medicina, Santiago, Chile; ^3^ Department of Cell Physiology and Molecular Biophysics, and Center for Membrane Protein Research, School of Medicine, Texas Tech University Health Sciences Center, Lubbock, TX, United States

**Keywords:** connexin, hemichannel, gap junction channel, ion channel, S-nitrosylation, phosphorylation, carbonylation

## Abstract

Connexins are a family of proteins that can form two distinct types of channels: hemichannels and gap junction channels. Hemichannels are composed of six connexin subunits and when open allow for exchanges between the cytoplasm and the extracellular milieu. Gap junction channels are formed by head-to-head docking of two hemichannels in series, each one from one of two adjacent cells. These channels allow for exchanges between the cytoplasms of contacting cells. The lens is a transparent structure located in the eye that focuses light on the retina. The transparency of the lens depends on its lack of blood irrigation and the absence of organelles in its cells. To survive such complex metabolic scenario, lens cells express Cx43, Cx46 and Cx50, three connexins isoforms that form hemichannels and gap junction channels that allow for metabolic cooperation between lens cells. This review focuses on the roles of Cx46 hemichannels and gap junction channels in the lens under physiological conditions and in the formation of cataracts, with emphasis on the modulation by posttranslational modifications.

## Introduction

The eye lens is a small but important organ that focuses light on the retina, which is essential for clear vision. The lens cells are subjected to a hard metabolic environment as they live under hypoxic conditions and do not have organelles. Cell-to-cell communication allows for the metabolic cooperation between lens cells through the fluxes of inorganic ions, small organic molecules and water (Goodenough et al., 1980; Mathias et al., 1997; Harris and Locke, 2009; Valiunas et al., 2018), with the lens operating as a syncytium of cells interconnected by gap junction channels formed by proteins called connexins (Goodenough, 1992; Giannone et al., 2021).

There is a strong link between connexin mutations and the appearance of cataracts (clouding of the lens). Due to the importance of Cx46 (a connexin isoform) in lens physiology (Jiang, 2010; Berthoud and Ngezahayo, 2017; Bai et al., 2021), and the recent discovery of its importance in cancer (Banerjee et al., 2010; Burr et al., 2011; Mulkearns-Hubert et al., 2019; Acuña et al., 2020; Acuña et al., 2021; Orellana et al., 2021), our group have been interested in the posttranslational regulation of this protein, and this review focuses on how Cx46 posttranslational regulation affects lens cells’ functions.

## Connexins in the Lens

**FIGURE 1:**
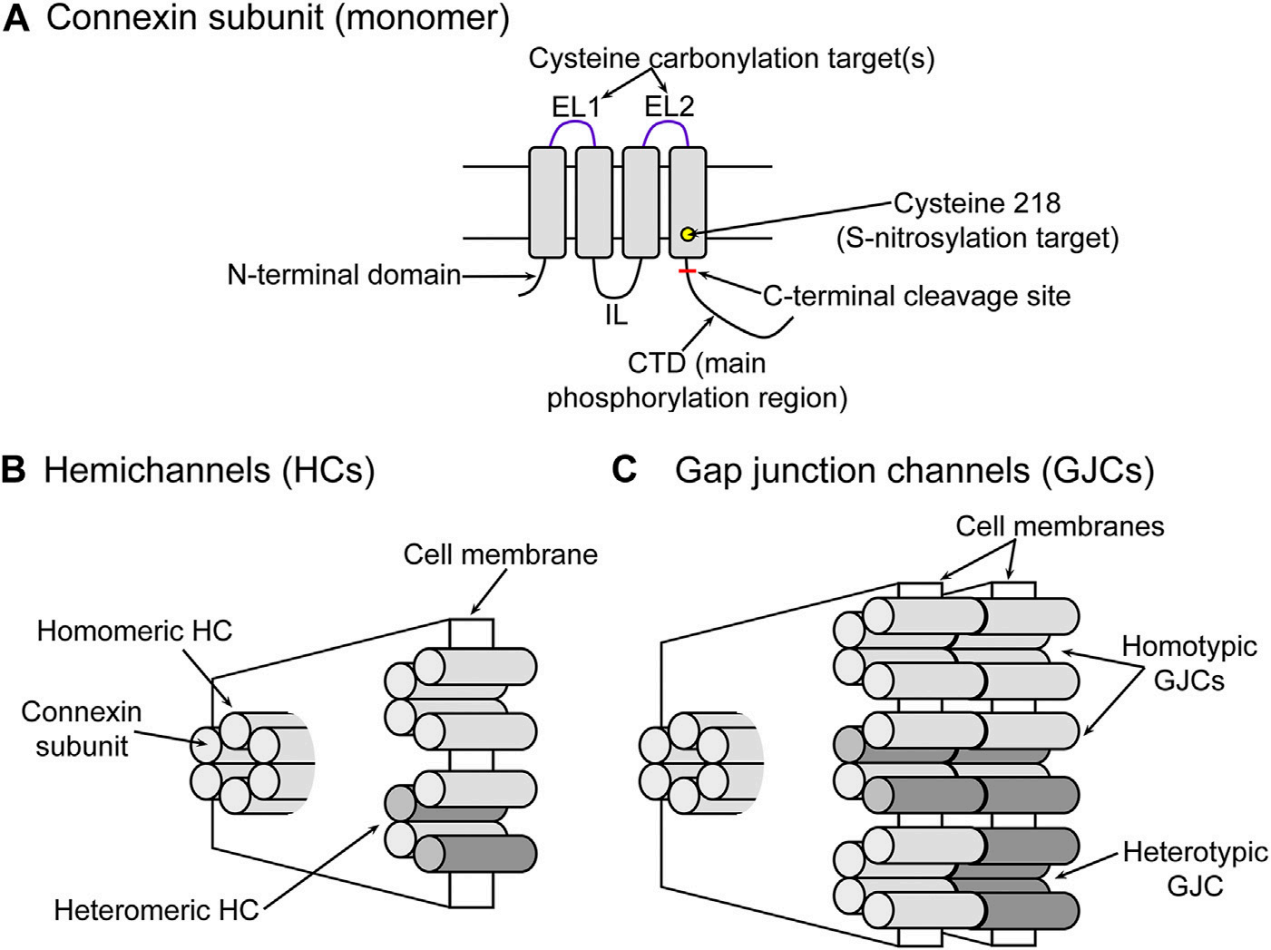
Connexins, hemichannels and gap junction channels. **(A)** Schematic representation of a connexin subunit. EL1 and EL2, extracellular loops; IL, intracellular loop; CTD, C-terminal domain. The rectangle blocks represent transmembrane helices 1–4. The location targets of some of the posttranslational modifications discussed in the text (phosphorylation, S-nitrosylation and carbonylation) are indicated. **(B)** Schematic representation of a connexin hemichannel. Each cylinder corresponds to a connexin subunit as seen in panel **(A)**. The two different tones represent different connexin isoforms (e.g., Cx46 and Cx50) and indicate hemichannels formed by only one isoform (homomeric) or more than one (heteromeric). **(C)** Schematic representation of a gap junction channel. Gap junction channels can be formed by docking of two hemichannels of the same subunit composition (homotypic) or hemichannels of different composition (heterotypic).

Gap junction channels are formed by head-to-head docking of two hemichannels in series, each from one of two adjacent cells (Saez et al., 2003; Mese et al., 2007; Nielsen et al., 2012; Abascal and Zardoya, 2013), where each hemichannel is formed by oligomerization of six connexin subunits (Saez et al., 2003; Mese et al., 2007; Nielsen et al., 2012; Abascal and Zardoya, 2013). Even though gap junction channels and hemichannels are formed by the same proteins (Figure 1), they have very different roles in cell physiology. Gap junction channels and hemichannels allow the permeation of ions and molecules up to 1.5 kDa, but while gap junction channels mediate exchanges between cytoplasms of adjacent cells, hemichannels do so between the intracellular and extracellular fluids, which have very different compositions (Harris and Locke, 2009; Vinken, 2015; Valiunas et al., 2018).

**FIGURE 2:**
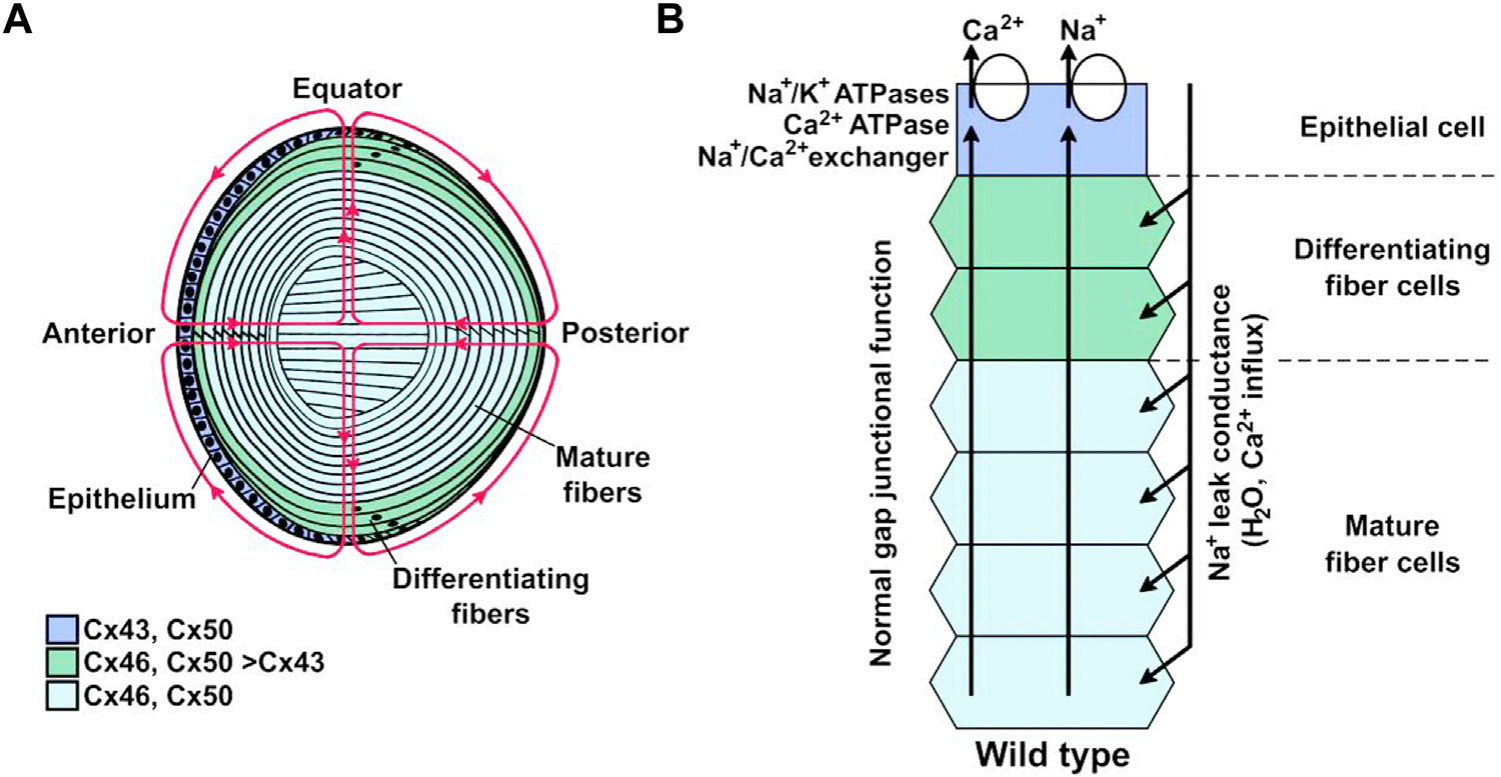
Lens internal circulation. **(A)** Schematic representation of the lens and its internal circulation. Ions and fluid enter the extracellular spaces at the anterior and posterior poles and leave through the epithelial cell membranes at the equator. The colors indicate the expression of the predominant connexin isoforms in different regions. Cells from the anterior epithelium express Cx43 and Cx50; differentiating fiber cells express Cx43, Cx46, and Cx50; mature fiber cells express Cx46 and Cx50. **(B)** Representation of the ion circulation and some of the components that support ion movements from the lens core to its surface. Na^+^ and Ca^2+^ that enter the lens at the anterior and posterior poles move to the center through the extracellular spaces where influx into fiber cells is driven by their electrochemical gradients across the membranes. They move towards the lens surface at the equator through gap junction channels, as the coupling conductance is highest at the equator, where these ions are extruded from the epithelial cells. Ion movements generate transmembrane osmotic gradients that couple their movement to that of fluid. Reproduced with permission from the Int. J. Med. Sci. 21(16):5822 (Berthoud et al., 2020).

There are 21 human genes coding for different connexin isoforms, which are expressed in almost all cell types (Sohl and Willecke, 2004; Abascal and Zardoya, 2013). Among them, Cx23 and Cx43 have been identified mostly in lens epithelial and differentiating fiber cells, while Cx46 and Cx50 are predominantly located in mature fiber cells (see Figure 2A) (Puk et al., 2008; Berthoud and Beyer, 2009; Beyer and Berthoud, 2014). Relevant to the lens pathology, mutations in Cx46 and Cx50 have been associated to lens malfunction and cataract formation (Beyer and Berthoud, 2014; Retamal et al., 2015; Berthoud et al., 2020; Bai et al., 2021). In particular for Cx46, it has been reported that mice lacking this connexin develop cataracts due to the absence of Cx46-based communication between mature fiber cells and more peripheral cells (Gong et al., 1998) and that Cx46 mutations that result in non-functional gap junction channels (*e.g.*, N63S and frame-shift mutant fs380) produce cataracts in human lenses (Pal et al., 2000; Berthoud and Ngezahayo, 2017; Berthoud et al., 2020). In summary, the current data show that the presence of cell-to-cell communication mediated by Cx46 gap junction channels is fundamental for the normal function and transparency of the lens.

The lens is a biconvex structure of the eye whose main function is to focus light on the retina. To fulfill this task the lens must be transparent, a characteristic achieved by several factors that include the absence of blood vessels, lack of organelles and tight packing of mature fiber cells. The lens consists of the anterior surface epithelium and the fiber cells that form the bulk of the organ (Figure 2A). The differentiating fiber cells arise from the epithelial cells in the equatorial region, which transform into mature fiber cells by a process that involves cell elongation and loss of organelles, including the nuclei. In the absence of blood vessels, survival of the fiber cells depends on an internal circulation system that allows delivery of ions and nutrients, and removal of waste metabolites to and from the center of the lens, respectively (Mathias et al., 2007; Berthoud et al., 2020). Lens circulation depends on communication through intercellular gap junction channels (Dawes et al., 2014; Berthoud and Ngezahayo, 2017; Berthoud et al., 2020; Giannone et al., 2021). Since Cx46 seems to be the most important connexin in fiber lens cells, its properties define the intracellular circuit properties in the lens (Mathias et al., 2007; Berthoud et al., 2020). Consistent with a critical role of Cx46 gap junction channels in lens circulation, there is a flux of glutathione (GSH) from the outer cortex to the mature fiber cells at the lens center, and GSH in the lens center is reduced in Cx46-but not in Cx50-knockout mice (Slavi et al., 2014). Figure 2 shows a representation of the lens and its circulation system. More details can be found in published reviews (Mathias et al., 2007; Berthoud et al., 2020).

### Characteristics of Gap Junction Channels and Hemichannels Formed by Cx46 and Their Roles in Lens Physiology

In the early 1990s, two independent groups localized the Cx46 gene (*GJA3*) in chromosome 13 (Hsieh et al., 1991; Mignon et al., 1996). Northern blot analyses revealed that Cx46 mRNA was highly expressed in rat lens and to a lesser degree in the heart, and was almost undetectable in the kidney (Paul et al., 1991). Cx46 mRNA distribution in mice is similar to that in rat, whereas in hamster Cx46 seems to be expressed only in the lens (Cruciani et al., 2004). In humans, according to the “Human Protein Atlas” (https:// www.proteinatlas.org/ ENSG00000121743-GJA3/tissue), Cx46 mRNA is present in the lens, heart, kidneys, and female and male reproductive organs. At a functional level, Cx46 can form gap junction channels and hemichannels (Paul et al., 1991; Ebihara and Steiner, 1993; Verselis et al., 2000), and also seems to have non-canonical functions such as in exosome-mediated communication (Acuña et al., 2020). As the lens is an avascular organ, lens cells live under hypoxia, and it has been proposed that Cx46 protects against its deleterious effects (Banerjee et al., 2010). Although the Cx46 promoter is activated by the hypoxia inducible factor in human epithelial lens cells in culture (Molina and Takemoto, 2012), this mechanism cannot operate *in vivo* as fiber cells lack a nucleus. Our review focuses on Cx46 as a channel-forming protein in the lens.

#### Cx46 Gap Junction Channels

Cx46 forms gap junction channels between fiber cells in bovine (Paul et al., 1991; Tenbroek et al., 1992), chicken (Biswas et al., 2010), mouse (Gong et al., 1998; Berthoud et al., 2016), and monkey (Lo et al., 1996) lenses. The current evidence based on the association of cataract development with Cx46 mutations strongly supports the notion that Cx46 also forms gap junction channels in human fiber cells (Jiang, 2010; Retamal et al., 2015; Berthoud and Ngezahayo, 2017; Berthoud et al., 2020). In most studies, the gap junction channel conductance was in the 130 to 180 pS range, but higher values have also been reported (*e.g.*, ∼250 pS in mice fiber cells) (Donaldson et al., 1995; Hopperstad et al., 2000; Sakai et al., 2003; Rubinos et al., 2014; Yue et al., 2021). The reason for the differences in single-channel conductance are unknown, but they may be due to the formation of heterogeneous channels (heteromeric hemichannels containing different connexin isoforms and/or heterotypic channels formed by hemichannels of different subunit composition) (Figure 1).

Gap junction channels display two distinct voltage-gating phenomena that are intrinsic properties of the hemichannels, fast (or trans-junctional voltage) gating, and slow (or loop) gating (Trexler et al., 1996; Bukauskas and Peracchia, 1997; Verselis, 2000; Verselis et al., 2000; Bukauskas et al., 2002; Bukauskas and Verselis, 2004; Gonzalez et al., 2007; Bargiello and Brink, 2009; Bargiello et al., 2012; Bargiello et al., 2018). Closure by slow-gating is favored by inside-negative voltages in hemichannels formed by all connexin isoforms, whereas the polarity of the fast gating varies among connexin isoforms (Verselis, 2000; Verselis et al., 2000; Harris, 2001; Bukauskas et al., 2002; Bukauskas and Verselis, 2004; Gonzalez et al., 2007; Bargiello et al., 2018). The fast gating occurs by transitions between the open and subconductance states (Trexler et al., 1996; Bukauskas and Verselis, 2004; Gonzalez et al., 2007; Bargiello et al., 2018), whereas in the slow gating a series of small amplitude transitions appear as a slow and complete closure (Trexler et al., 1996; Bukauskas and Verselis, 2004; Gonzalez et al., 2007; Bargiello et al., 2018). It is believed that the slow-gating mechanism also mediates “chemical” gating in response to changes in intracellular Ca^2+^ and pH, as well as hemichannel opening during gap junction formation (loop gating) (Bukauskas and Peracchia, 1997; Bukauskas and Verselis, 2004). Our knowledge of the structure and location of the voltage sensors and gates is complex and details can be found elsewhere (Harris, 2001; Bukauskas and Verselis, 2004; Gonzalez et al., 2007; Bargiello et al., 2012; Bargiello et al., 2018; Natha et al., 2021).

**FIGURE 3:**
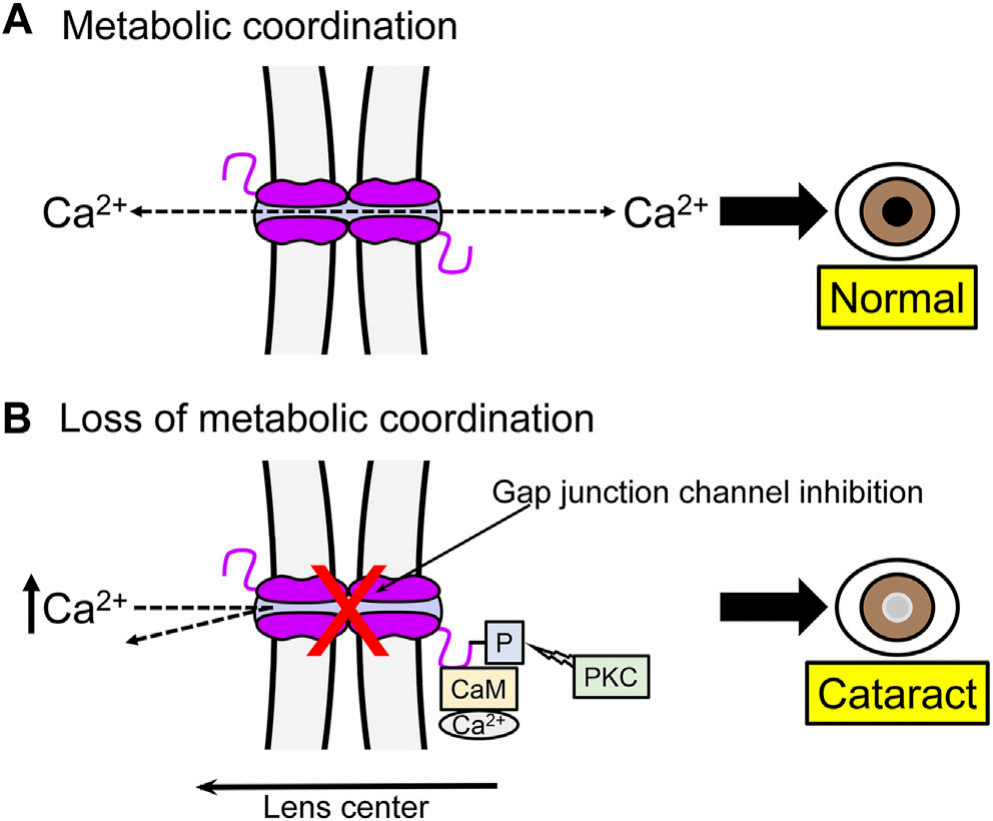
One example of the role of gap junction channels in metabolic coordination between lens fiber cells. **(A)** Normal junctional coupling. Under normal conditions gap junction communication prevents Ca^2+^ accumulation in lens fibers. The Ca^2+^ transported from the central to peripheral cells is extruded by the Ca^2+^ pump and a Na^+^/Ca^2+^ exchanger. **(B)** Loss of gap junctional coupling. Inhibition of gap junction communication can lead to Ca^2+^ accumulation and development of cataracts. Factors such as phosphorylation (-P) of Cx46 and association with Ca^2+^-calmodulin (CaM) can inhibit gap junction channels.

Under normal conditions intracellular Ca^2+^ flows from the center to the periphery of the lens, where the Ca^2+^-ATPase and Na^+^/Ca^2+^ exchangers transport it out of the lens, avoiding its accumulation (Figure 2) (Gao et al., 2004). Consistent with this, the lack of Cx46 in mice lens fiber cells results in development of nuclear cataracts associated to crystallin proteolysis by caspases (Gong et al., 1997) and Lp82 (Baruch et al., 2001), both Ca^2+^-dependent proteases. A possible explanation is that the absence of Cx46 reduces Ca^2+^ flow through Cx46 gap junction channels, which leads to Ca^2+^ accumulation in the cytoplasm of fiber cells, followed by protease activation (Figure 3) (Gao et al., 2004). When Cx46 was knocked into both Cx50 alleles in mice lenses, fiber cells homeostasis and gap junction coupling were maintained, but the size of the lens was reduced (White, 2002; Martinez-Wittinghan et al., 2004). Therefore, gap junction channels mediate metabolic coupling, but intercellular channels formed by Cx46 and Cx50 are not fully exchangeable. Potentially relevant to this, gap junction channels formed by Cx43, Cx46 and Cx50 are permeable to second messengers, but permeation rates vary, with cAMP permeability of Cx46 gap junction channels much higher than that of Cx50 channels, although both are lower than that of Cx43 channels (Qu and Dahl, 2002; Brink et al., 2020).

#### Cx46 Hemichannels

Formation of Cx46 hemichannels in living cells was first suggested when overexpression of Cx46 in *Xenopus* oocytes resulted in cell lysis, correlated with Lucifer yellow uptake, a large outward current, and plasma membrane depolarization (Paul et al., 1991). *Xenopus laevis* oocytes expressing Cx46 show large currents that are usually activated slowly (time constant in the tens seconds) at voltages above +5–10 mV (Ebihara and Steiner, 1993; Trexler et al., 1996; Retamal et al., 2010). Cx46 hemichannels are cation selective and show marked rectification, with conductances of ∼300 and ∼135 pS at −50 mV and +50 mV, respectively (Trexler et al., 1996). In addition to the main conductance of ∼300 pS at negative membrane voltages, Cx46 hemichannels display a sub-conductance of 100–200 pS (Trexler et al., 1996; Pfahnl and Dahl, 1998).

Hemichannels are not only activated by membrane depolarization but are also modulated by extracellular Ca^2+^. In the normal mM range this ion maintains Cx46 hemichannels mostly closed (Verselis and Srinivas, 2008) and this inhibition is modulated by membrane voltage (Pfahnl and Dahl, 1999) and monovalent ions (Srinivas et al., 2006). Studies on Cx26 hemichannels showed that Ca^2+^ produces subtle conformational changes near the pore’s extracellular entrance (Bennett et al., 2016; Lopez et al., 2016). It appears that Ca^2+^ disrupts an intersubunit salt bridge, which results in neighboring electrostatic interactions that involve other charged residues (Lopez et al., 2016), and it has been proposed that a network of electrostatic intrasubunit and intersubunit interactions plays a critical role in hemichannel gating, with two anionic residues in this region interacting with Ca^2+^ directly to occlude the pore (Lopez et al., 2016). Recent work on Cx46 hemichannels showed that the voltage sensor movement and Ca^2+^ binding domain are allosterically coupled, and it has been proposed that Ca^2+^ binds to negative charges in the voltage sensor that are reachable by Ca^2+^ only in the inside-negative voltage resting conformation of the voltage sensor (Pinto et al., 2017). Cx46 hemichannels also present a characteristic that we called “facilitation,” which refers to the increase in current in response to repetitive positive voltage pulses (> +60 mV), a phenomenon dependent on the time between pulses and the extracellular Ca^2+^ concentration (Retamal et al., 2010). Thus, Cx46 hemichannel properties are complex and are modulated by small molecules normally presents in the extracellular medium.

Cx46 hemichannels are also mechanosensitive (Bao L. et al., 2004), with hemichannels’ activity increasing when negative pressure is applied to the recording pipette or in response to cell swelling by exposure to hypotonic solution (Bao L. et al., 2004). It has been suggested that the mechanosensitivity of Cx46 hemichannels has a role in fluid equilibration after mechanical stresses associated with the change in shape of the lens during the process of accommodation to focus light on the retina from objects at different distances.

Although the presence of functional connexin hemichannels in the lens can be inferred from studies in the 1990s (Rae et al., 1992; Rae and Rae, 1992; Eckert et al., 1998), demonstration that lens fiber cells present currents that can be linked to gap junction channels and hemichannels formed by Cx46 was obtained 20 years later in freshly dissociated fiber cells from Cx50 knockout mouse lenses (Ebihara et al., 2011). In this study, Cx46 hemichannels currents activated by depolarization and removal of extracellular Ca^2+^ displayed a large conductance and allowed propidium uptake from the extracellular space (Ebihara et al., 2011). Despite their low open probability at resting potential, the activity of Cx46 hemichannels seems sufficient to account for a constant Na^+^ entry into the fiber cells that depolarizes the membrane potential by a few mV (Ebihara et al., 2014), which may an essential part of the Na^+^ lens circuit flux (Figure 2). Moreover, Cx46 hemichannel activity enhances Cx46 gap junction channel formation in *Xenopus* oocytes (Beahm and Hall, 2004), and therefore, there could be a balance between Cx46 hemichannel activity and gap junction channel formation in lens fiber cells.

**FIGURE 4:**
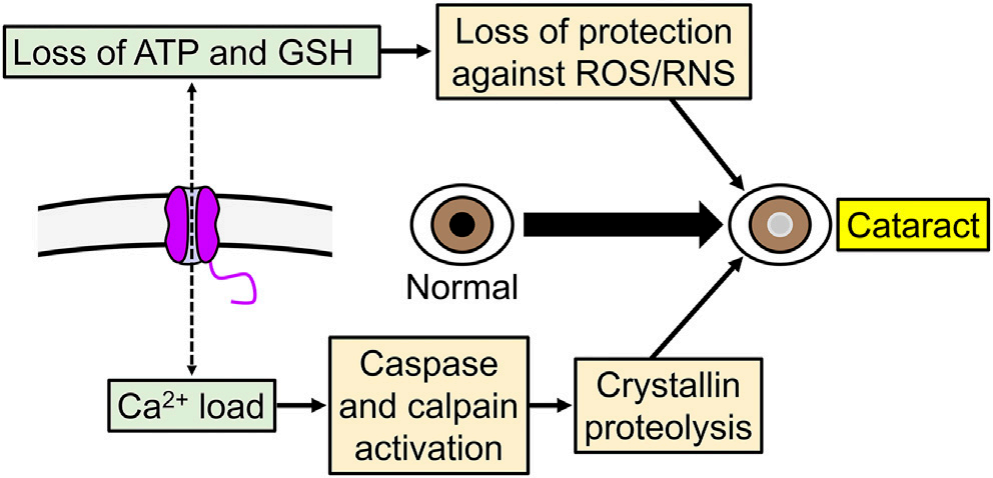
Example of the role of increased Cx46 hemichannel activity in cataracts development. Abnormal increase in hemichannel activity can produce a variety of potentially deleterious effects in cells, including increases in Ca^2+^ influx, and ATP and glutathione (GSH) efflux.

Since a low level of hemichannel activity may be responsible for the chronic Na^+^ current in the normal lens (Ebihara et al., 2014) and may also participate in cell volume regulation (Bao L. et al., 2004), reduced opening could affect accommodation or lead to cataracts by affecting the internal circulation of the lens. Conversely, increased hemichannel opening can lead to the development of cataracts due to damaging disbalances that result from changes that include increases in Na^+^ and Ca^2+^ influxes, losses of K^+^, ATP and GSH, and depolarization (Figure 4). Hyperactive hemichannels can be the result of mutations or a variety of mechanisms that include membrane depolarization, increases in intracellular Ca^2+^, and changes in the phosphorylation and redox status (Bao L. et al., 2004; Bukauskas and Verselis, 2004; Saez et al., 2005; Berthoud and Beyer, 2009; Fasciani et al., 2013; Retamal et al., 2015; Figueroa et al., 2019; Berthoud et al., 2020; Peracchia, 2020; Natha et al., 2021; Retamal et al., 2021). Overall, the information available supports a role of Cx46 hemichannels in the lens. The electrical and permeability behaviors of Cx46 hemichannels are complex and more studies *in vivo* are needed to understand the role of Cx46 hemichannels in lens physiology and pathophysiology.

## Cx46 Posttranslational Modifications and Their Potential Relevance to Lens Physiology

### Phosphorylation

This is a reversible posttranslational modification in which a protein kinase adds a phosphate group to serine, threonine, or tyrosine residues, which can be removed by protein phosphatases. The phosphorylation status of a protein can affect expression and/or function and it is frequently an important regulator (Cohen, 2002; Fischer, 2013), as is the case for gap junction channels and hemichannels (Saez et al., 1998; Lampe and Lau, 2000, 2004; Moreno, 2005; Pogoda et al., 2016). Western blot analyses of bovine and rat lenses showed that Cx46 migrates as two prominent bands of ∼46 kDa and 56–60 kDa (Paul et al., 1991), and analyses of cultured bovine lens revealed that the ∼46-kDa band corresponds to unphosphorylated Cx46 and the slower migrating bands to Cx46 phosphorylated at serine residues (Jiang et al., 1993). The degree of phosphorylation changes during development, with unphosphorylated Cx46 prevailing during early gestation and fully phosphorylated Cx46 after birth (Jiang et al., 1993). PKCγ is one of the kinases that regulates Cx46 phosphorylation status *in vivo*. It has been shown that Cx46 co-immunoprecipitates with PKCγ (Lewis et al., 2001; Saleh et al., 2001) and that PKCγ co-immunoprecipitation with Cx46 increases in rats lens exposed to oxidation by H_2_O_2_ (Lin et al., 2004). Under the latter condition, both serine and threonine Cx46 residues were phosphorylated (Lin et al., 2004). In contrast, another study showed that the activation of PKCγ by 12-O-tetradecanoylphorbol-13-acetate (TPA) only increased Cx46 phosphorylation of threonine residues (Zampighi et al., 2005). One explanation for the discrepancy is a high phosphorylation level of Cx46 serines under control conditions in the latter study.

It has been suggested that phosphorylation of Cx46 by PKC has a role in the development of cataracts associated with diabetes and oxidative stress, but the relationship between Cx46 phosphorylation and cataracts is not straightforward and likely depends on many factors. Diabetes is correlated with cataract development in humans and animal models, and in rats fed with high galactose (diabetes model) an increase in lens Cx46 serine phosphorylation by PKCγ and inhibition of dye transfer between fiber cells were observed (Lewis et al., 2001). In contrast, lenses from rats exposed to selenite, which causes cataract development due to an excess of free radicals, showed dephosphorylation of Cx46 forming gap junction channels (Fleschner, 2006). Consistent with the association of a decrease in Cx46 phosphorylation and cataracts, the absence PKCγ in a knockout mice was correlated with the development of cataracts and an increase of oxidative stress, suggesting that the lack of Cx46 regulation may disrupt channel function and make the cells prone to damage by oxidative stress (Lin et al., 2006).

A Mass spectrometry study determined 11 phosphorylation sites, all in the Cx46 C-terminal domain (Wang and Schey, 2009). However, hemichannels made of a C-terminal truncated form of rat Cx46 expressed in *Xenopus* oocytes were still inactivated by PKC stimulation with TPA (Walter et al., 2008). This result suggests the presence of Cx46 regulatory proteins phosphorylated by PKC and/or that PKC phosphorylates amino acids in the intracellular loop, or less likely the N-terminus, that were not identified in the Wang and Schey’s work (Wang and Schey, 2009). In addition to PKCγ, Cx46 has three putative target sites for phosphorylation by casein kinase 2 (CK2), and it seems that this kinase phosphorylates the Cx46 C-terminus and increases hemichannel formation (Walter et al., 2008). Contrary to CK2, neither PI3k nor Akt seem to modulate Cx46 gap junction channels expressed in *Xenopus* oocytes (Martinez et al., 2015).

In summary, phosphorylation by PKC and other kinases affects Cx46 expression and function, and cataract formation is associated with phosphorylation of Cx46 in diabetes and its dephosphorylation following oxidate stress. However, details on the role of Cx46 modifications in the pathophysiological mechanisms leading to the formation of cataracts are still unresolved. A summary of the main posttranslational modifications discussed in this review is presented in Table 1.

**TABLE 1:** Posttranslational modifications of Cx46.

Posttranslational modification	Target	Enzyme/molecule	Effects on GJCs	Effects on HCs	Reference
Phosphorylation	Ser	ND	ND	ND	Jiang et al. (1993)
	Ser and Thr	PKCγ	ND	ND	Lin et al. (2004)
	Thr	PKCγ	ND	ND	Zampighi et al. (2005)
	Ser	PKCγ	Dye transfer	ND	Lewis et al. (2001)
	Ser348,Ser410	PKCγ	ND	ND	Walter et al. (2008)
	Thr307	CK2	ND	HC formation	Walter et al. (2008)
S-nitrosylation	Cys	NO	ND	Voltage inactivation	Retamal et al. (2009)
	Cys218	NO	No effects	Activation	Retamal et al. (2019)
Carbonylation	Extracellular Cys	4-HNE	ND	Inhibition	Retamal et al. (2020)
	Extracellular Cys	CO	ND	Inhibition	Leon-Paravic et al. (2014)
Cleavage	Leu255	—	Dye transfer	ND	Jacobs et al. (2004)
	Ile238-P251		ND	ND	Slavi et al. (2016)

The table shows a summary of posttranslational modifications presented in the text. See references for the Cx46 ortholog studied and additional details. GJC: gap junction channel; HC: hemichannel; ND: not determined; CK2: casein kinase 2; NO: nitric oxide; 4-HNE: 4-hydroxynonenal; CO: carbon monoxide.

### S-Nitrosylation

Nitric oxide (NO) is a gaseous transmitter that signals through the activation of the guanylyl cyclase and the formation of S-nitrosothiols, resulting in S-nitrosylated proteins (Hess et al., 2005; Nakamura and Lipton, 2016; Lee et al., 2021). NO is a short-lived and highly reactive gas produced during conversion of arginine to citrulline and NO by NO synthases (Snyder, 1992; Iwanaga et al., 1999), and NO production is stimulated by Ca^2+^ or regulated at the transcriptional level, depending on the synthases involved (Bredt and Snyder, 1994; Nelson et al., 2003; Nakamura and Lipton, 2016). The formation of S-nitrosylated proteins results from reaction between a redox-sensitive thiol group (actually a thiolate anion) and a nitrosonium cation (NO^+^) in the presence of transition metals that accept an electron from NO (Hess et al., 2005; Martinez-Ruiz et al., 2011; Nakamura and Lipton, 2016; Lee et al., 2021), although other mechanisms are possible (Hess et al., 2005; Martinez-Ruiz et al., 2011; Smith and Marletta, 2012; Nakamura and Lipton, 2016), including protein-to-protein transnitrosylation. In the latter, NO is transferred from a donor protein to a specific acceptor protein (Nakamura and Lipton, 2013; Jia et al., 2014). Not all cysteine thiols in a protein can be S-nitrosylated. Although details on the S-nitrosylation selectivity are not completely understood, S-nitrosylation is promoted by proximity to the NO source and local hydrophobicity that serves to concentrate NO (*e.g.*, thiols close to the membrane), the presence of charged amino acids in close proximity, and location in flexible regions (Liu et al., 1998; Hess et al., 2005; Doulias et al., 2010; Smith and Marletta, 2012; Nakamura and Lipton, 2016; Lee et al., 2021). S-nitrosylation is often a relatively labile modification and reducing agents and enzymes such as thioredoxin and S-nitrosoglutathione (GSNO) reductases can remove the NO from the modified cysteines (Benhar et al., 2008; Benhar et al., 2009; Nakamura and Lipton, 2016).

An association between connexins, redox potential and oxidative stress is well established (Quan et al., 2021; Zhang et al., 2021), although low or physiological levels NO could have a protective effect in lens epithelial cells (Chamberlain et al., 2008). A high production of NO in human lens has been associated with cataracts, especially in hypertensive patients (Ornek et al., 2003) and smokers (Anitha et al., 2019), and a similar association has been found in animal models of hypertension and diabetes (Abdul Nasir et al., 2017; Yadav et al., 2018). For example, rats injected with CdCl_2_ develop hypertension and lens opacity, and the lens opacity was diminished by topical application of the NO synthases inhibitor L-N^G^-Nitro arginine methyl ester and was aggravated by the NO donor S-nitrosoglutathione (Yadav et al., 2018). The deleterious effect of NO on lens cells could be the result of the decreases of membrane transport efficiency, intracellular ATP, and/or GSH observed in rat lenses after exposition to a NO donor (Varma and Hegde, 2007). Additionally, a decrease of cytochrome c oxidase expression and function was found in a rat model of cataract where an increase of NO production was observed (Nagai and Ito, 2007).

Based on the association between NO and cataracts, we studied the effects of NO donors on Cx46 hemichannels and found that following exposure to S-nitrosoglutathione Cx46 hemichannels expressed in *Xenopus* oocytes opened at lower positive voltages, displayed a current inactivation evident at voltages above +50 mV, and showed increased tail currents (Retamal et al., 2009). These changes did not occur in hemichannels formed by a Cx46 mutant without cysteines at positions 218 (transmembrane segment 4 near membrane/cytosol interface), 283 and 321 (C-terminus), or in oocytes treated with the reducing agent dithiothreitol (DTT), suggesting that one or more of the mutated cysteines becomes S-nitrosylated by NO. We identified cysteine 218 as the NO sensor and found that, unexpectedly, NO did not affect Cx46 gap junction channels (Retamal et al., 2019). These observations suggest that cysteine 218 is not available for modification in the gap junction channels or that conformational changes that follow hemichannel docking prevent the effect of S-nitrosylation. We also found that rats injected with selenite developed cataracts and that Cx46 was S-nitrosylated in the lenses extracted from the selenite-treated animals (Retamal et al., 2019). In addition, exposure to NO donors increased hemichannel activity as measured by an increase of ethidium uptake in human lens epithelial cells in culture (HLE-B3 cells). This rise in hemichannel activity was correlated with an increase of S-nitrosylated Cx46 as determined by immunoprecipitation and Western blot analyses (Retamal et al., 2019). In summary, Cx46 hemichannels are sensitive to NO, which modifies cysteine 218. S-nitrosylation affects Cx46 hemichannel properties, which could play a role in cataract development and/or could worsen the effects of signaling pathways activated in diseases such as diabetes.

### Carbonylation

Protein carbonylation is a posttranslational modification in which amino acids are chemically modified by free radicals through a non-enzymatic reaction, and where oxidative stress is often involved (Wong et al., 2013a; Hwang et al., 2016; Tola et al., 2021). Under normal conditions, there is a balance between the generation of reactive oxygen species (ROS) and antioxidant defenses. Primary carbonylation consist of the modification of amino acids such as lysine, proline and threonine by free radicals to form ketones and aldehydes (Tola et al., 2021), whereas secondary carbonylation is associated with the oxidation of nucleophilic amino acids by lipid peroxides (cysteine > histidine > lysine) (Wong et al., 2013a; Zhang and Forman, 2017). The latter can be reversed in a thiol-dependent manner (Wong et al., 2013b). Reactive carbonyl species produced by oxidation of polyunsaturated fatty acids and sugars are highly reactive and can easily carbonylate proteins (Uchida et al., 1999; Basta et al., 2004; Hwang et al., 2016; Gaschler and Stockwell, 2017). The lipid peroxidation product 4-hydroxinonenal (4-HNE) is one of the most abundant and toxic reactive carbonyl species, which is generated *via* β-cleavage of hydroperoxide derived from ω-6 polyunsaturated fatty acids such as linoleic acid and arachidonic acid (Uchida et al., 1999; Carini et al., 2004; Hwang et al., 2016; Zhang and Forman, 2017).

Unsaturated fatty acids are hydrophobic molecules that have multiple roles in physiological and pathological conditions, and their excess is associated with the development of cataracts (Iwig et al., 2004). With this in mind, we tested the effects of linoleic acid (an omega-3 essential fatty acid) on Cx46 hemichannels. At low concentration (0.1 μM) linoleic acid increased hemichannel currents in *Xenopus* oocytes by a process that requires activation of an intracellular mechanism (Retamal et al., 2011). However, at concentrations above 1 μM the fatty acid inhibited Cx46 hemichannels in a dose-dependent manner (Retamal et al., 2011). The inhibitory effect was PKC- and intracellular Ca^2+^-independent but depended on the integrity of the linoleic acid double bond at position 9 (Retamal et al., 2011). As for the case of S-nitrosylation (Retamal et al., 2019), linoleic acid did not affect Cx46 gap junction channels (Retamal et al., 2011). A simple explanation for these results is that linoleic acid and other fatty acids are oxidized to lipid peroxides, which are much more reactive than the non-oxidized lipids. In this context, the prevention of the increase of lipid peroxides precluded lens opacification in an animal model of cataracts (Nagai et al., 2008). Also, consistent with this notion, the lipid peroxidation product 4-HNE also inhibits Cx46 hemichannels in a dose dependent manner and its effect is reversed by DTT, suggesting the involvement of cysteine oxidation (Retamal et al., 2020). Western blot analysis revealed that Cx46 expressed in HeLa cells becomes carbonylated after exposure to 4-HNE, and that DTT reduced this carbonylation (Retamal et al., 2020). Additional studies with Cx46 cysteine mutants suggested that the inhibitory effect of 4-HNE on hemichannels occurs through carbonylation of extracellular cysteines, which could explain the absence of effects on gap junction channels (Retamal et al., 2011), as these cysteines are not available for modification in the latter (Foote et al., 1998; Maeda et al., 2009; Myers et al., 2018). This is congruent with the importance of extracellular cysteines in gap junction channel formation due to the formation of disulfide bridges (Bao X. et al., 2004; Maeda et al., 2009; Heja et al., 2021), whereas Cx43 without cysteines can still form functional hemichannels (Bao X. et al., 2004). The potential role of carbonylation is stressed by the finding of Cx46 carbonylation in rat’s lenses with cataracts (Retamal et al., 2020). It seems therefore possible that peroxidation products of fatty acids oxidation can inhibit Cx46 hemichannels through carbonylation of extracellular cysteines and that this effect may lead to cataract formation.

Carbon monoxide (CO) is a metabolite produced by decomposition of heme groups by heme oxygenases (Yang et al., 2021). In general, CO protects against oxidative stress (Yang et al., 2021) and exogenous administration of CO-donors has been used for the treatment of conditions associated with oxidative stress, such as inflammation, sepsis, lung and cardiovascular diseases, and cancer (Bauer and Pannen, 2009; Motterlini and Otterbein, 2010; Ryter and Choi, 2013). In lens epithelial cells in culture the expression of heme oxygenase-1 (HO-1) increased in response to oxidative stress (Padgaonkar et al., 1997) and the cell damage elicited by H_2_O_2_ was inhibited by the CO-releasing molecule 3 (CORM-3) through the inhibition of NF-κB nuclear translocation, reduction of ROS concentration, and enhancement of GSH and superoxide dismutase levels (Huang et al., 2018). Accordingly, mice expressing a negative dominant HO-1 display accumulation of lipid peroxides, reduced concentration of GSH, and develop cataracts (Huang et al., 2021). We found that CORM-2 produced a major decrease in the amplitude of the currents of Cx46 hemichannels expressed in *Xenopus laevis* oocytes (Leon-Paravic et al., 2014). The effect of the CO donor was independent on the presence of the Cx46 C-terminal domain but required the presence of extracellular Cx46 cysteines and was reversed by reducing agents. We also found that CORM-2 induces carbonylation of purified Cx46 (Leon-Paravic et al., 2014), but the amino acids modified have not been identified. Based on these observations, we have proposed that CO carbonylates extracellular Cx46 cysteines through lipid peroxides, which reduces Cx46 hemichannel activity, with a protective effect against oxidative stress and cataract development (Retamal, 2016).

The evidence presented above on the relationship between Cx46 hemichannel inhibition, carbonylation, and cataracts can be confusing, as increased carbonylation induced by 4-HNE is associated with hemichannel inhibition and cataracts, whereas CO is also associated with hemichannel inhibition but has a protective effect on the lens. As mentioned in *Characteristics of Gap Junction Channels and Hemichannels Formed by Cx46 and Their Roles in Lens Physiology* under Cx46 hemichannels, a normal hemichannel activity may be essential for the internal lens circulation, whereas abnormally high hemichannel activity can damage lens cells because of ionic and metabolic disbalances. Therefore, it is possible that the same posttranslational modification affects the lens differently, depending on the underlying hemichannel activity and experimental conditions, additional effects of fatty acids/4-HNE and CO, residues carbonylated, or differences in the signaling cascades involved in response to increases in fatty acids/4-HNE and CO.

### C-Terminal Cleavage

In early 2000s it was demonstrated that the C-terminal domain of native Cx46 of fiber cells can be cleaved. Cleavage was correlated with the loss of cell nuclei, decrease of gap junction plaques’ size and transfer of fluorescein between fiber cells (Jacobs et al., 2004). Using MS/MS mass spectrometry it was demonstrated that Cx44 (bovine orthologue of human Cx46) was cleaved at Leucine 255, while human Cx46 was cleaved between amino acids 238 and 251, and that Cx46 truncation increases with age (Slavi et al., 2016). As Cx46 gap junctions are blocked at intracellular acidic pH (Trexler et al., 1999) and the pH at the frog lens center is ∼6.8, a significant decrease of gap junction communication mediated by Cx46 can be expected in the center of the lens. However, fiber cells located at the lens center remain coupled upon acidification by increasing CO_2_ (Mathias et al., 1991). Therefore, it seems likely that C-terminus truncation maintains metabolic coupling between lens fibers in a pH independent way as fiber lens cells have the truncated version of Cx46 and remain ∼80% functional at pH 6.8 (Eckert, 2002).

It has been proposed for Cx40 and Cx43 that the C-terminal domain acts as a “ball” (“particle”) of a “ball-and-chain” (“particle-receptor”) pH-dependent gating mechanism where the C-terminal domain acts as gating particle that binds to a receptor in the cytoplasmic loop (Ek-Vitorin et al., 1996; Stergiopoulos et al., 1999; Anumonwo et al., 2001; Delmar et al., 2004; Hirst-Jensen et al., 2007; Ponsaerts et al., 2010; Oh and Bargiello, 2015; Khan et al., 2020). However, the role of the ball-and-chain mechanism in Cx46 pH gating is unclear as Cx46 gap junction channels truncated at amino acid 251 are still inhibited by acidification in HeLa cells (Slavi et al., 2016). This work suggests that the sensitivity of C-terminus truncated Cx46 to an acidic environment is cell dependent and/or that there are other mechanisms involved in addition to the ball-and-chain gating. In studies of mice lenses *ex vivo*, it was found that the pH sensitivity of Cx46 gap junction channels is complex and depends on the activity of Cx50, as the gap junction channels become insensitive to acidification in the presence of mefloquine, a selective Cx50 gap junction channel inhibitor (Martinez-Wittinghan et al., 2006). In summary, Cx46 is pH sensitive, but the gating mechanism is complex and more experiments are needed to unravel the roles of the truncation in lens physiology and pathophysiology.

### Others

Oxidative stress has a great impact in lens physiology and it is well established that an excess of free radicals is associated with the development of cataracts (Berthoud and Beyer, 2009). The work of glutathione peroxidase-1 (GPRx-1 or GSHPx-1) is one of the ways that lens cells use to fight against the excess of free radicals. This enzyme is a catalase that reduces H_2_O_2_ bioavailability and therefore protect lens cells from the damage induced by this molecule (Reddy et al., 2001). Knockout mice lacking this enzyme develop cataracts at early age that progress to complete opacification in older animals (Reddy et al., 2001). In this knockout animal model, the expression of Cx46 and Cx50 was reduced to half and the cell-to-cell conductance was reduced in outer differentiated fibers cells and inner core mature fibers, suggesting decreased gap junctional communication by oxidation as a factor in the development of cataracts in this model (Wang et al., 2009). It is interesting to note that gap junction coupling between fiber cells decreases with age, a phenomenon correlated with a decrease of Cx46 and Cx50 (Gao et al., 2013; Gong et al., 2021), which is associated with oxidative stress (Gao et al., 2013; Gong et al., 2021). We found that whole-cell Cx46 hemichannel currents recorded from freshly obtained *Xenopus* oocytes increase progressively, but as time passes inactivation at positive voltages (> +50 mV) tends to appear (Retamal et al., 2019). We believe that this inactivation arises from the oxidation of the channel, which could have consequences such as changes in its permeability to large molecules (Retamal et al., 2009). In summary, oxidation can reduce both expression and function of gap junction channels and hemichannels formed by Cx46. The reduction of gap junction coupling by an increase in oxidative stress could be explained by a reduction in Cx46 content and/or some of the posttranslational modifications described above.

Protein-protein interactions also modulate connexin channels and hemichannels (Sorgen et al., 2018; Van Campenhout et al., 2020). It has been reported that Cx46 in the lens interacts with Ca^2+^-calmodulin (CaM) and that a Cx46 mutant (G143R) associated with cataracts in humans enhances the Cx46-CaM interaction and forms gap junction channels and hemichannels with altered voltage gating and permeability (Hu et al., 2018). From these results, it has been suggested that the alteration in Cx46-CaM interaction can mediate the deleterious effect of some Cx46 mutations in lens fiber cells (Figure 3) (Hu et al., 2020).

## Summary

Cx46 forms functional hemichannels and gap junction channels in lens fiber cells. These channels participate in different cell tasks, and their malfunction favors cataracts formation. Hemichannels and gap junction channels formed by different connexin isoforms are controlled by posttranslational modifications. In the case of Cx46 channels, they are modulated by a variety of factors, including phosphorylation, oxidation, carbonylation, and C-terminal cleavage. Pathological conditions can diminish or enhance molecular pathways associated to these modifications and alter Cx46-based channel properties, which in turn can produce ion and metabolites disbalances. Therefore, understanding the molecular mechanisms of Cx46 regulation could benefit future new therapies against cataracts (Retamal et al., 2021).
